# Aqua­bis(3′-hydr­oxy-2,2′-bipyridine-3-olato-κ^2^ *N*,*N*′)zinc(II)

**DOI:** 10.1107/S1600536808024495

**Published:** 2008-08-06

**Authors:** Shi Guo Zhang, Chao Hou

**Affiliations:** a Department of Chemistry and Chemical Engineering, Institute of Materials Chemistry, Binzhou University, Binzhou 256603, People’s Republic of China; b Department of Chemistry, Shandong Normal University, Jinan 250014, People’s Republic of China

## Abstract

In the title complex, [Zn(C_10_H_7_N_2_O_2_)_2_(H_2_O)], the Zn^II^ ion and water O atom are located on a crystallographic twofold rotation axis and the metal atom assumes a distorted trigonal-bipyramidal ZnN_4_O coordination geometry. An intra­molecular O—H⋯O hydrogen bond occurs within the ligand and inter­molecular O—H⋯O hydrogen bonds involving the water mol­ecule result in a sheet structure in the crystal structure. In addition, a short C—O⋯π contact between the O atom of the deprotonated hydroxyl group and a nearby pyridine ring [O⋯*Cg* = 3.977 (2) Å, where *Cg* is the centroid of the pyridine ring] is observed.

## Related literature

For related structures, see: Cargill Thompson *et al.* (1996 ▶); Stephenson & Hardie (2007 ▶).
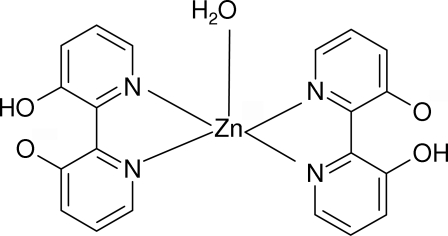


## Experimental

### 

#### Crystal data

[Zn(C_10_H_7_N_2_O_2_)_2_(H_2_O)]
*M* *_r_* = 457.74
Orthorhombic, 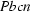

*a* = 13.931 (2) Å
*b* = 9.1685 (16) Å
*c* = 14.364 (3) Å
*V* = 1834.7 (6) Å^3^
*Z* = 4
Mo *K*α radiation
μ = 1.38 mm^−1^
*T* = 298 (2) K
0.40 × 0.21 × 0.20 mm

#### Data collection

Bruker SMART APEX CCD diffractometer
Absorption correction: multi-scan (*SADABS*; Bruker, 1997 ▶) *T*_min_ = 0.608, *T*_max_ = 0.770
8883 measured reflections
1621 independent reflections
1293 reflections with *I* > 2σ(*I*)
*R*_int_ = 0.035

#### Refinement

*R*[*F* ^2^ > 2σ(*F* ^2^)] = 0.030
*wR*(*F* ^2^) = 0.083
*S* = 1.05
1621 reflections
137 parameters
H-atom parameters constrained
Δρ_max_ = 0.24 e Å^−3^
Δρ_min_ = −0.22 e Å^−3^

### 

Data collection: *SMART* (Bruker, 1997 ▶); cell refinement: *SAINT* (Bruker, 1997 ▶); data reduction: *SAINT*; program(s) used to solve structure: *SHELXTL* (Sheldrick, 2008 ▶); program(s) used to refine structure: *SHELXTL*; molecular graphics: *SHELXTL*; software used to prepare material for publication: *SHELXTL* and local programs.

## Supplementary Material

Crystal structure: contains datablocks I, global. DOI: 10.1107/S1600536808024495/hb2736sup1.cif

Structure factors: contains datablocks I. DOI: 10.1107/S1600536808024495/hb2736Isup2.hkl

Additional supplementary materials:  crystallographic information; 3D view; checkCIF report

## Figures and Tables

**Table 1:** Selected bond lengths (Å)

Zn1—N1	2.081 (2)
Zn1—N2	2.0716 (18)
O3—Zn1	2.002 (2)

**Table 2:** Hydrogen-bond geometry (Å, °)

*D*—H⋯*A*	*D*—H	H⋯*A*	*D*⋯*A*	*D*—H⋯*A*
O1—H5⋯O2	0.82	1.61	2.410 (3)	165
O3—H1⋯O2^i^	0.80	1.83	2.630 (2)	174

Symmetry code: (i) 
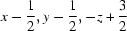
.

**Fig. 1.**
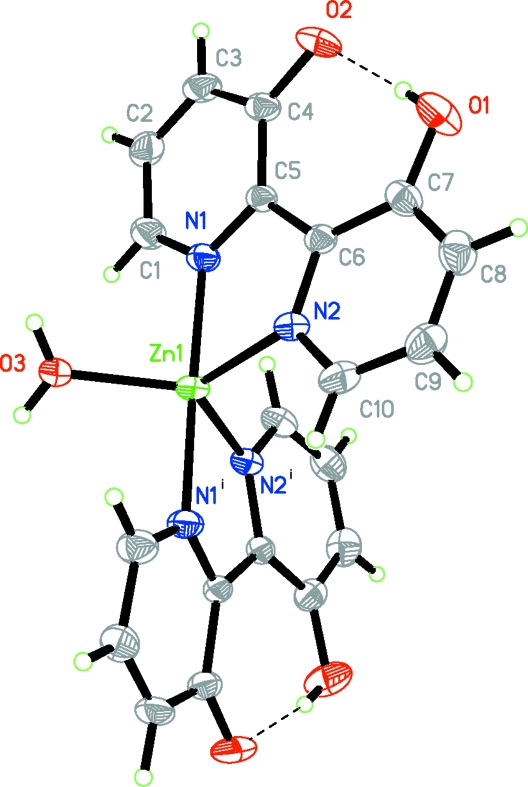
View of (I), showing displacement ellipsoids for the non-hydrogen atoms drawn at the 30% probability level. The hydrogen bond is indicated by a dashed line. Symmetry code: (i) -1/2 + x, -1/2 + y, 3/2 - z

**Fig. 2.**
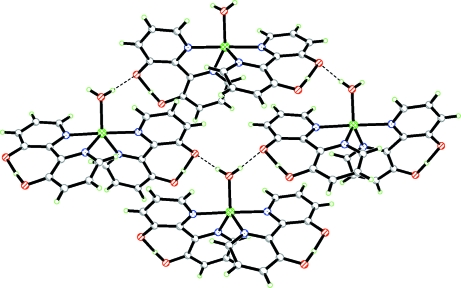
Intermolecular hydrogen bonds (dashed line) in the crystal of (I).

## supplementary crystallographic information

### Comment

2,2'-Bipyridine-3,3'-diol (bpd) has the potential to be a useful multi-dentate ligand and may act as a bridging ligand due to coordination of its N and O atoms.  A few multi-nuclear complexes have been synthesized with mono-deprotonated bpd as a bridging ligand (Cargill Thompson *et al.* 1996; Stephenson & Hardie, 2007). We attempted to prepare a similar compound, but instead we obtained the mono-nuclear title complex, (I), (Fig. 1) and report its structure herein.

The Zn1 atom in (I) lies in a twofold axis and is in a distorted trigonal bipyramidal geometry (Table 1).  The dihedral angle between the N1 and N2 ring mean planes is 13.08 (12)°. An intramolecular O—H···O hydrogen bond between O atom of deprotonated hydroxyl and hydroxyl group (Table 2) occcurs within the ligand.  The water molecule (O atom site symmetry 2) makes an intermolecular O—H···.O hydrogen bond to result in a supramolecular sheet structure in the *ab* plane (Fig. 2). In addition to the hydrogen bonds there is weak interaction between C—O bond and pyridine ring and the relevant distances are as follows: C4—O2···*Cg*1^i^ = 3.977 (2) Å and C4—O2···*Cg*1^i^_perp_ = 3.635 Å [*Cg*1 is the centroid of N1/C1—C5 ring, C4—O2···*Cg*1^i^_perp_ is the perpendicular distance from O2 atom to ring *Cg*1^i^; symmetry code: (i) 1/2-*x*, 1/2+*y*, *z*].

### Experimental

A 10-ml H_2_O solution of ZnCl_2_ (0.1214 g, 0.891 mmol) was added to a 10-ml hot ethanol solution containing 2,2'-bipyridine-3,3'-diol (0.1664 g, 0.884 mmol), and the mixed solution was stirred for a few minutes. Yellow blocks of (I) were obtained after the solution had been allowed to stand at room temperature for two weeks.

### Refinement

The water H atom was located in a difference Fourier map and refined as riding in its as found relative position with *U*_iso_(H) = 1.5*U*_eq_(O). The other H atoms geometrically placed (C—H = 0.93-0.96Å, O—H = 0.82Å) and refined as riding with with *U*_iso_ = *U*_iso_ = 1.2*U*_eq_(C) or 1.5*U*_eq_(methyl C, O).

### Crystal data

**Table:**

[Zn(C_10_H_7_N_2_O_2_)_2_(H_2_O)]	*F*_000_ = 936
*M**_r_* = 457.74	*D*_x_ = 1.657 Mg m^−^^3^
Orthorhombic, *P**b**c**n*	Mo *K*α radiation λ = 0.71073 Å
Hall symbol: -P 2n 2ab	Cell parameters from 2299 reflections
*a* = 13.931 (2) Å	θ = 2.7–24.6º
*b* = 9.1685 (16) Å	µ = 1.38 mm^−^^1^
*c* = 14.364 (3) Å	*T* = 298 (2) K
*V* = 1834.7 (6) Å^3^	Block, yellow
*Z* = 4	0.40 × 0.21 × 0.20 mm

### Data collection

**Table:**

Bruker SMART APEX CCD diffractometer	1621 independent reflections
Radiation source: fine-focus sealed tube	1293 reflections with *I* > 2σ(*I*)
Monochromator: graphite	*R*_int_ = 0.035
*T* = 298(2) K	θ_max_ = 25.0º
φ and ω scans	θ_min_ = 2.7º
Absorption correction: multi-scan(SADABS; Bruker, 1997)	*h* = −15→16
*T*_min_ = 0.608, *T*_max_ = 0.770	*k* = −9→10
8883 measured reflections	*l* = −17→17

### Refinement

**Table:**

Refinement on *F*^2^	Secondary atom site location: difference Fourier map
Least-squares matrix: full	Hydrogen site location: difmap and geom
*R*[*F*^2^ > 2σ(*F*^2^)] = 0.030	H-atom parameters constrained
*wR*(*F*^2^) = 0.083	*w* = 1/[σ^2^(*F*_o_^2^) + (0.0435*P*)^2^ + 0.4048*P*] where *P* = (*F*_o_^2^ + 2*F*_c_^2^)/3
*S* = 1.05	(Δ/σ)_max_ < 0.001
1621 reflections	Δρ_max_ = 0.24 e Å^−^^3^
137 parameters	Δρ_min_ = −0.22 e Å^−^^3^
Primary atom site location: structure-invariant direct methods	Extinction correction: none

### Special details

**Table:**

Geometry. All e.s.d.'s (except the e.s.d. in the dihedral angle between two l.s. planes) are estimated using the full covariance matrix. The cell e.s.d.'s are taken into account individually in the estimation of e.s.d.'s in distances, angles and torsion angles; correlations between e.s.d.'s in cell parameters are only used when they are defined by crystal symmetry. An approximate (isotropic) treatment of cell e.s.d.'s is used for estimating e.s.d.'s involving l.s. planes.
Refinement. Refinement of *F*^2^ against ALL reflections. The weighted *R*-factor *wR* and goodness of fit *S* are based on *F*^2^, conventional *R*-factors *R* are based on *F*, with *F* set to zero for negative *F*^2^. The threshold expression of *F*^2^ > σ(*F*^2^) is used only for calculating *R*-factors(gt) *etc*. and is not relevant to the choice of reflections for refinement. *R*-factors based on *F*^2^ are statistically about twice as large as those based on *F*, and *R*- factors based on ALL data will be even larger.

### Fractional atomic coordinates and isotropic or equivalent isotropic displacement parameters (Å^2^)

**Table:**

	*x*	*y*	*z*	*U*_iso_*/*U*_eq_	
C1	0.18495 (17)	−0.0831 (3)	0.84200 (18)	0.0497 (6)	
H8A	0.1443	−0.1318	0.8829	0.060*	
C2	0.28226 (18)	−0.0979 (3)	0.85235 (18)	0.0505 (6)	
H7	0.3078	−0.1560	0.8993	0.061*	
C3	0.34041 (17)	−0.0251 (3)	0.7919 (2)	0.0484 (6)	
H6A	0.4066	−0.0346	0.7980	0.058*	
C4	0.30414 (16)	0.0627 (3)	0.72164 (17)	0.0405 (6)	
C5	0.20250 (14)	0.0718 (2)	0.71420 (15)	0.0328 (5)	
C6	0.14629 (15)	0.1528 (2)	0.64177 (14)	0.0335 (5)	
C7	0.18121 (17)	0.2589 (3)	0.57966 (15)	0.0433 (6)	
C8	0.1158 (2)	0.3273 (3)	0.52075 (16)	0.0532 (7)	
H4	0.1371	0.4013	0.4816	0.064*	
C9	0.0216 (2)	0.2884 (3)	0.51908 (18)	0.0530 (7)	
H3	−0.0212	0.3329	0.4783	0.064*	
C10	−0.00846 (16)	0.1818 (3)	0.57930 (17)	0.0482 (6)	
H2	−0.0724	0.1528	0.5782	0.058*	
N1	0.14707 (14)	−0.00122 (19)	0.77522 (15)	0.0401 (5)	
N2	0.05150 (12)	0.11824 (19)	0.63953 (12)	0.0379 (5)	
O1	0.27183 (13)	0.3017 (2)	0.57387 (13)	0.0673 (6)	
H5	0.3044	0.2553	0.6111	0.101*	
O2	0.36348 (11)	0.1306 (2)	0.66513 (12)	0.0574 (5)	
O3	0.0000	−0.2225 (3)	0.7500	0.0640 (8)	
H1	−0.0443	−0.2657	0.7730	0.096*	
Zn1	0.0000	−0.00413 (4)	0.7500	0.03787 (16)	

### Atomic displacement parameters (Å^2^)

**Table:**

	*U*^11^	*U*^22^	*U*^33^	*U*^12^	*U*^13^	*U*^23^
C1	0.0382 (14)	0.0538 (16)	0.0571 (16)	−0.0007 (11)	−0.0022 (12)	0.0162 (12)
C2	0.0420 (14)	0.0485 (15)	0.0610 (16)	0.0054 (11)	−0.0147 (12)	0.0037 (12)
C3	0.0248 (12)	0.0561 (16)	0.0644 (16)	0.0022 (11)	−0.0092 (13)	−0.0119 (13)
C4	0.0291 (12)	0.0451 (13)	0.0474 (13)	−0.0045 (11)	0.0016 (11)	−0.0133 (11)
C5	0.0264 (11)	0.0330 (12)	0.0389 (12)	−0.0026 (9)	0.0007 (9)	−0.0086 (10)
C6	0.0311 (12)	0.0340 (11)	0.0353 (11)	0.0003 (9)	0.0039 (9)	−0.0069 (9)
C7	0.0451 (14)	0.0455 (14)	0.0393 (13)	−0.0082 (11)	0.0066 (11)	−0.0059 (11)
C8	0.0669 (19)	0.0496 (16)	0.0432 (14)	−0.0014 (13)	0.0054 (13)	0.0075 (12)
C9	0.0555 (16)	0.0570 (17)	0.0464 (15)	0.0136 (14)	−0.0026 (12)	0.0049 (13)
C10	0.0356 (14)	0.0567 (16)	0.0525 (15)	0.0114 (11)	−0.0028 (11)	0.0013 (13)
N1	0.0276 (11)	0.0446 (12)	0.0482 (11)	0.0002 (8)	0.0001 (9)	0.0067 (9)
N2	0.0272 (10)	0.0421 (11)	0.0444 (11)	0.0025 (8)	0.0014 (8)	0.0001 (8)
O1	0.0538 (12)	0.0821 (14)	0.0661 (12)	−0.0260 (10)	0.0022 (9)	0.0184 (11)
O2	0.0277 (9)	0.0776 (13)	0.0669 (11)	−0.0141 (8)	0.0075 (8)	−0.0009 (10)
O3	0.0353 (14)	0.0391 (14)	0.118 (2)	0.000	0.0278 (13)	0.000
Zn1	0.0232 (2)	0.0418 (3)	0.0487 (3)	0.000	0.00383 (15)	0.000

### Geometric parameters (Å, °)

**Table:**

C1—N1	1.328 (3)	C7—C8	1.392 (3)
C1—C2	1.370 (3)	C8—C9	1.361 (4)
C1—H8A	0.9300	C8—H4	0.9300
C2—C3	1.362 (4)	C9—C10	1.371 (4)
C2—H7	0.9300	C9—H3	0.9300
C3—C4	1.386 (4)	C10—N2	1.336 (3)
C3—H6A	0.9300	C10—H2	0.9300
C4—O2	1.315 (3)	O1—H5	0.8200
C4—C5	1.422 (3)	O3—H1	0.8042
C5—N1	1.346 (3)	Zn1—N1	2.081 (2)
C5—C6	1.499 (3)	Zn1—N2	2.0716 (18)
C6—N2	1.359 (3)	Zn1—N2^i^	2.0716 (18)
C6—C7	1.407 (3)	Zn1—N1^i^	2.081 (2)
C7—O1	1.324 (3)	O3—Zn1	2.002 (2)
			
N1—C1—C2	121.8 (2)	C8—C9—C10	118.0 (2)
N1—C1—H8A	119.1	C8—C9—H3	121.0
C2—C1—H8A	119.1	C10—C9—H3	121.0
C3—C2—C1	118.1 (2)	N2—C10—C9	121.9 (2)
C3—C2—H7	121.0	N2—C10—H2	119.0
C1—C2—H7	121.0	C9—C10—H2	119.0
C2—C3—C4	122.1 (2)	C1—N1—C5	121.6 (2)
C2—C3—H6A	118.9	C1—N1—Zn1	120.67 (16)
C4—C3—H6A	118.9	C5—N1—Zn1	117.24 (15)
O2—C4—C3	119.7 (2)	C10—N2—C6	121.4 (2)
O2—C4—C5	123.5 (2)	C10—N2—Zn1	121.03 (15)
C3—C4—C5	116.8 (2)	C6—N2—Zn1	116.41 (14)
N1—C5—C4	119.5 (2)	C7—O1—H5	109.5
N1—C5—C6	113.49 (18)	Zn1—O3—H1	119.5
C4—C5—C6	126.9 (2)	O3—Zn1—N2	122.79 (5)
N2—C6—C7	118.8 (2)	O3—Zn1—N2^i^	122.79 (5)
N2—C6—C5	114.11 (18)	N2—Zn1—N2^i^	114.42 (10)
C7—C6—C5	127.03 (19)	O3—Zn1—N1	90.73 (5)
O1—C7—C8	116.9 (2)	N2—Zn1—N1	77.61 (7)
O1—C7—C6	125.0 (2)	N2^i^—Zn1—N1	101.58 (7)
C8—C7—C6	118.1 (2)	O3—Zn1—N1^i^	90.73 (5)
C9—C8—C7	121.6 (2)	N2—Zn1—N1^i^	101.58 (7)
C9—C8—H4	119.2	N2^i^—Zn1—N1^i^	77.61 (7)
C7—C8—H4	119.2	N1—Zn1—N1^i^	178.53 (10)
			
N1—C1—C2—C3	0.3 (4)	C4—C5—N1—Zn1	−172.45 (15)
C1—C2—C3—C4	0.3 (4)	C6—C5—N1—Zn1	5.8 (2)
C2—C3—C4—O2	−180.0 (2)	C9—C10—N2—C6	−2.3 (4)
C2—C3—C4—C5	−1.0 (4)	C9—C10—N2—Zn1	165.07 (19)
O2—C4—C5—N1	−179.9 (2)	C7—C6—N2—C10	0.6 (3)
C3—C4—C5—N1	1.2 (3)	C5—C6—N2—C10	179.53 (19)
O2—C4—C5—C6	2.1 (4)	C7—C6—N2—Zn1	−167.35 (15)
C3—C4—C5—C6	−176.8 (2)	C5—C6—N2—Zn1	11.6 (2)
N1—C5—C6—N2	−11.3 (3)	C10—N2—Zn1—O3	102.57 (17)
C4—C5—C6—N2	166.8 (2)	C6—N2—Zn1—O3	−89.42 (15)
N1—C5—C6—C7	167.5 (2)	C10—N2—Zn1—N2^i^	−77.43 (17)
C4—C5—C6—C7	−14.4 (3)	C6—N2—Zn1—N2^i^	90.58 (15)
N2—C6—C7—O1	−179.3 (2)	C10—N2—Zn1—N1	−174.67 (19)
C5—C6—C7—O1	1.9 (4)	C6—N2—Zn1—N1	−6.65 (14)
N2—C6—C7—C8	2.2 (3)	C10—N2—Zn1—N1^i^	4.08 (19)
C5—C6—C7—C8	−176.6 (2)	C6—N2—Zn1—N1^i^	172.10 (14)
O1—C7—C8—C9	178.0 (2)	C1—N1—Zn1—O3	−48.31 (19)
C6—C7—C8—C9	−3.4 (4)	C5—N1—Zn1—O3	123.54 (16)
C7—C8—C9—C10	1.8 (4)	C1—N1—Zn1—N2	−171.8 (2)
C8—C9—C10—N2	1.1 (4)	C5—N1—Zn1—N2	0.05 (16)
C2—C1—N1—C5	−0.1 (4)	C1—N1—Zn1—N2^i^	75.4 (2)
C2—C1—N1—Zn1	171.39 (19)	C5—N1—Zn1—N2^i^	−112.72 (16)
C4—C5—N1—C1	−0.7 (3)	C1—N1—Zn1—N1^i^	131.69 (18)
C6—C5—N1—C1	177.6 (2)	C5—N1—Zn1—N1^i^	−56.46 (16)

Symmetry codes: (i) −*x*, *y*, −*z*+3/2.

### Hydrogen-bond geometry (Å, °)

**Table:**

*D*—H···*A*	*D*—H	H···*A*	*D*···*A*	*D*—H···*A*
O1—H5···O2	0.82	1.61	2.410 (3)	165
O3—H1···O2^ii^	0.80	1.83	2.630 (2)	174

Symmetry codes: (ii) *x*−1/2, *y*−1/2, −*z*+3/2.
